# Admission uric acid to HDL-C ratio and long-term mortality after hip fracture in older adults: a retrospective cohort study

**DOI:** 10.3389/fmed.2026.1906596

**Published:** 2026-10-21

**Authors:** Shuang Han, Ya-Hui Fu, Chao Ke, Bin-Fei Zhang

**Affiliations:** 1 Department of Orthopedic Trauma, Honghui Hospital, Xi'an Jiaotong University, Xi’an, Shaanxi Province, China; 2 Department of Joint Surgery, Honghui Hospital, Xi'an Jiaotong University, Xi’an, Shaanxi Province, China

**Keywords:** elderly, high-density lipoprotein cholesterol, hip fractures, mortality, nonlinear relationship, prognostic biomarker, uric acid

## Abstract

**Objective:**

To investigate the association between the uric acid to high-density lipoprotein cholesterol (HDL-C) ratio (UHR) and long-term all-cause mortality in elderly patients with hip fractures.

**Methods:**

We retrospectively included patients aged 65 years or older who underwent surgery for hip fracture. Admission UHR was calculated from the first available serum uric acid and HDL-C measurements. The primary endpoint was all-cause mortality during follow-up. Cox regression was used for conventional adjusted analyses, and restricted cubic spline and two-piecewise Cox models were used as exploratory analyses of possible nonlinearity.

**Results:**

We included 304 patients (104 men and 200 women), with a mean age of 80.00 ± 6.50 years. During a mean follow-up of 34.42 ± 14.76 months, 82 patients died. In the fully adjusted conventional Cox models, UHR was not statistically significant when analyzed as a continuous variable (*P* = 0.1413) or by tertiles (high vs. low: HR = 1.54, 95% CI 0.84–2.84, *P* = 0.1624). In an exploratory two-piecewise model, the estimated inflection point was 166.17. Below this value, higher UHR was associated with higher mortality (HR per unit = 1.01, 95% CI 1.00–1.03, *P* = 0.0326), whereas the slope above the inflection point was not significant (*P* = 0.8050). The likelihood-ratio test comparing the single-slope and two-piecewise models yielded *P* = 0.037.

**Conclusion:**

Admission UHR showed an exploratory nonlinear association with long-term all-cause mortality in this single-center cohort. Because conventional fully adjusted analyses were not statistically significant and the inflection point was derived from the same dataset, UHR should not yet be considered an independent predictor or a validated clinical cutoff. External prospective validation with repeated measurements is required.

**Clinical Trial Registration:**

ChiCTR: ChiCTR2200057323.

## 1 Introduction

Hip fractures are one of the most severe osteoporotic fractures and have become a critical global public health issue due to their high disability rate, high mortality rate, and substantial healthcare economic burden. With the ongoing acceleration of population aging, the global incidence of hip fractures is expected to continue rising significantly over the coming decades (1–3). Despite continuous advancements in surgical techniques and perioperative management, elderly patients with hip fractures still face a series of serious challenges post-surgery, including poor functional recovery (4), various complications (such as deep vein thrombosis (5), infections (6), and cardiovascular events (7), and even death (8). Therefore, identifying biomarkers that can effectively and early predict patient outcomes is of crucial clinical significance for implementing risk stratification, optimizing clinical decision-making, and improving patient prognosis.

Currently, the assessment of prognosis in elderly patients with hip fractures primarily relies on traditional clinical indicators such as age and comorbidities (9, 10). However, these indicators often fail to reflect the patient's underlying pathophysiological state accurately. In recent years, researchers have gradually shifted their focus toward comprehensive biomarkers that can quantify the body's metabolic and inflammatory levels. Uric acid (UA) (11–13) and high-density lipoprotein cholesterol (HDL-C) ^(^14, 15) are two such indicators that have garnered significant attention. As an endogenous antioxidant, uric acid is closely associated with adverse outcomes in cardiovascular diseases (16), metabolic syndrome (17), and chronic kidney disease (18). On the other hand, high-density lipoprotein cholesterol is known for its vascular protective functions, including anti-inflammatory, antioxidant, and cholesterol reverse transport properties (19). It is noteworthy that the biological effects of these two markers in the body are not independent but involve complex interactions.

Based on this, recent research has proposed a composite indicator: the Uric acid to HDL-C Ratio (UHR). UHR is expected to simultaneously reflect the body's oxidative stress status and the level of dyslipidemia. In the field of cardiovascular disease, UHR has been proven to be a strong predictor of prognosis for conditions such as coronary heart disease and heart failure (20). However, the clinical value of this indicator in the field of traumatic orthopedics, particularly among the vulnerable elderly hip fracture population, has not yet been fully explored.

We hypothesized that admission UHR may capture part of the metabolic and inflammatory disturbance accompanying hip fracture and may be associated with postoperative mortality. We therefore explored both conventional and nonlinear associations between UHR and long-term all-cause mortality. Given the retrospective design and the use of a single post-injury measurement, the analysis was intended to generate hypotheses rather than establish causality or a clinical decision threshold.

## 2 Materials and methods

### 2.1 Study design and institutional review board approval

This retrospective cohort study was conducted at the trauma center of Honghui Hospital. Patient enrollment spanned from January 1, 2015, to September 30, 2019. The study protocol received approval from the hospital's Ethics Committee (Approval No. 202201009), which waived the need for informed consent due to the retrospective design. The reporting of this study adheres to the STROCSS 2021 guidelines (21).

### 2.2 Participants

Patient demographic and clinical data were retrieved from medical records. Eligibility required patients to be aged ≥65 years, with a radiologically confirmed femoral neck or intertrochanteric fracture, who underwent surgical treatment at our hospital, and had complete hospitalization data. The sole exclusion criterion was being inaccessible for follow-up via telephone to determine survival status.

### 2.3 Hospital treatment

All patients were diagnosed with hip fractures via x-ray upon admission. The primary surgical management modalities, determined by fracture type and patient profile, included closed reduction internal fixation (CRIF), open reduction internal fixation (ORIF), hemiarthroplasty (HA), and total hip arthroplasty (THA).

### 2.4 Variables

This study assessed demographic, clinical, and surgical variables, including age, sex, injury mechanism, fracture classification, age-adjusted Charlson Comorbidity Index (aCCI, points), specific comorbidities, treatment strategy, time to admission (hours), time to operation (days), operation time (minutes), blood loss (mL), transfusion (units), infusion volume (mL), length of stay (days), and follow-up duration (months). Admission UHR was calculated as serum uric acid (μmol/L) divided by HDL-C (mmol/L) using the first laboratory measurements obtained after admission. Because this numerical ratio depends on the units used, the value of 166.17 is specific to this unit convention. The primary endpoint was all-cause mortality during follow-up.

### 2.5 Follow-up

After discharge, family members of patients were contacted by telephone to record their survival status and the time elapsed since discharge.

### 2.6 Statistical analysis

Continuous variables were expressed as mean ± standard deviation (SD), while categorical variables were summarized as frequencies and percentages. Table 1 provides descriptive comparisons of baseline characteristics across UHR tertiles using the *χ*^2^ test, one-way ANOVA, Kruskal–Wallis H test, or Fisher exact test, as appropriate. These between-tertile *P* values were not used for covariate selection. Candidate covariates were identified from the univariable Cox analysis in Table 2 based on *P* < 0.10 and from established clinical relevance. Age, aCCI, CHD, arrhythmia, and dementia met the statistical criterion; sex was included *a priori* because of its recognized prognostic importance after hip fracture.

**Table 1:** Baseline characteristics according to UHR tertiles.

UHR tertile	Low	Medium	High	*P*-value	*P*-value*
N	100	101	103		
UHR	126.83 ± 26.09	208.27 ± 26.06	353.22 ± 91.45	<0.001	<0.001
Age (years)	79.01 ± 6.13	80.25 ± 6.79	80.73 ± 6.50	0.153	0.147
Sex				<0.001	-
Male	18 (18.00%)	38 (37.62%)	48 (46.60%)		
Female	82 (82.00%)	63 (62.38%)	55 (53.40%)		
Injury mechanism				0.47	0.581
Falling	98 (98.00%)	98 (97.03%)	100 (97.09%)		
Accident	1 (1.00%)	1 (0.99%)	3 (2.91%)		
Other	1 (1.00%)	2 (1.98%)	0 (0.00%)		
Fracture classification				0.042	-
Intertrochanteric fracture	61 (61.00%)	76 (75.25%)	77 (74.76%)		
Femoral neck fracture	39 (39.00%)	25 (24.75%)	26 (25.24%)		
aCCI	4.16 ± 1.12	4.37 ± 1.14	4.36 ± 1.05	0.321	0.417
Hypertension				0.32	-
No	48 (48.00%)	41 (40.59%)	39 (37.86%)		
Yes	52 (52.00%)	60 (59.41%)	64 (62.14%)		
Diabetes				0.793	-
No	82 (82.00%)	79 (78.22%)	83 (80.58%)		
Yes	18 (18.00%)	22 (21.78%)	20 (19.42%)		
CHD				0.013	-
No	63 (63.00%)	43 (42.57%)	57 (55.34%)		
Yes	37 (37.00%)	58 (57.43%)	46 (44.66%)		
Arrhythmia				0.544	-
No	77 (77.00%)	71 (70.30%)	77 (74.76%)		
Yes	23 (23.00%)	30 (29.70%)	26 (25.24%)		
Hemorrhagic stroke				0.879	1
No	98 (98.00%)	99 (98.02%)	100 (97.09%)		
Yes	2 (2.00%)	2 (1.98%)	3 (2.91%)		
Ischemic stroke				0.658	-
No	69 (69.00%)	64 (63.37%)	66 (64.08%)		
Yes	31 (31.00%)	37 (36.63%)	37 (35.92%)		
Cancer				0.187	0.233
No	99 (99.00%)	96 (95.05%)	101 (98.06%)		
Yes	1 (1.00%)	5 (4.95%)	2 (1.94%)		
Associated injuries				0.347	-
No	96 (96.00%)	92 (91.09%)	97 (94.17%)		
Yes	4 (4.00%)	9 (8.91%)	6 (5.83%)		
Dementia				0.639	-
No	93 (93.00%)	97 (96.04%)	97 (94.17%)		
Yes	7 (7.00%)	4 (3.96%)	6 (5.83%)		
COPD				0.912	-
No	95 (95.00%)	96 (95.05%)	99 (96.12%)		
Yes	5 (5.00%)	5 (4.95%)	4 (3.88%)		
Hepatitis				0.614	0.789
No	98 (98.00%)	100 (99.01%)	100 (97.09%)		
Yes	2 (2.00%)	1 (0.99%)	3 (2.91%)		
Gastritis				0.348	0.317
No	96 (96.00%)	100 (99.01%)	101 (98.06%)		
Yes	4 (4.00%)	1 (0.99%)	2 (1.94%)		
Time to admission (hours)	66.49 ± 177.77	190.73 ± 576.39	83.06 ± 235.47	0.039	0.034
Time to operation (days)	4.30 ± 2.07	4.85 ± 2.43	4.87 ± 2.44	0.142	0.147
Treatment strategy				0.078	0.037
CRIF/ORIF	59 (59.00%)	75 (74.26%)	77 (74.76%)		
HA	40 (40.00%)	25 (24.75%)	26 (25.24%)		
THA	1 (1.00%)	1 (0.99%)	0 (0.00%)		
Operation time (minutes)	95.66 ± 27.88	95.41 ± 34.89	104.20 ± 44.04	0.146	0.377
Blood loss (mL)	222.47 ± 120.43	225.71 ± 126.88	239.80 ± 169.45	0.657	0.935
Transfusion (units)	0.97 ± 1.25	0.99 ± 1.12	1.12 ± 1.33	0.657	0.742
Infusion (mL)	1,553.33 ± 353.71	1,546.94 ± 348.25	1,636.77 ± 401.77	0.17	0.18
Length of stay (days)	8.01 ± 2.87	8.41 ± 2.63	8.81 ± 3.16	0.149	0.102
Follow-up (months)	36.24 ± 14.74	33.57 ± 16.48	33.48 ± 12.84	0.32	0.259
Mortality				0.046	-
Survival	82 (82.00%)	70 (69.31%)	70 (67.96%)		
Dead	18 (18.00%)	31 (30.69%)	33 (32.04%)		

*P* values compare baseline characteristics across UHR tertiles and are descriptive only; they were not used to select covariates for the multivariable Cox models. *P* values were calculated using one-way analysis of variance or the chi-square test. For continuous variables, *P* values marked with an asterisk (*) were calculated using the Kruskal-Wallis test. For categorical variables, Fisher's exact test was used when expected cell counts were less than 10.

UHR, uric acid to high-density lipoprotein cholesterol ratio; CHD, coronary heart disease; COPD, chronic obstructive pulmonary disease; HA, hemiarthroplasty; CRIF/ORIF, closed/open reduction and internal fixation; THA, total hip arthroplasty.

**Table 2:** Univariable Cox regression analysis for all-cause mortality.

Variables	Statistics	HR (95% CI)	*P*-value
Age (years)	80.00 ± 6.50	1.08 (1.04, 1.12)	<0.0001
Sex			
Male	104 (34.21%)	1	
Female	200 (65.79%)	0.73 (0.47, 1.14)	0.1668
Injury mechanism			
Falling	296 (97.37%)	1	
Accident	5 (1.64%)	0.66 (0.09, 4.72)	0.676
Other	3 (0.99%)	2.99 (0.73, 12.19)	0.1269
Fracture classification			
Intertrochanteric fracture	214 (70.39%)	1	
Femoral neck fracture	90 (29.61%)	0.86 (0.53, 1.41)	0.551
aCCI	4.30 ± 1.10	1.32 (1.09, 1.59)	0.004
Hypertension			
No	128 (42.11%)	1	
Yes	176 (57.89%)	0.83 (0.54, 1.28)	0.3967
Diabetes			
No	244 (80.26%)	1	
Yes	60 (19.74%)	1.18 (0.71, 1.97)	0.5316
CHD			
No	163 (53.62%)	1	
Yes	141 (46.38%)	1.63 (1.05, 2.53)	0.0284
Arrhythmia			
No	225 (74.01%)	1	
Yes	79 (25.99%)	1.58 (1.00, 2.50)	0.0491
Hemorrhagic stroke			
No	297 (97.70%)	1	
Yes	7 (2.30%)	0.00 (0.00, Inf)	0.9957
Ischemic stroke			
No	199 (65.46%)	1	
Yes	105 (34.54%)	0.95 (0.60, 1.51)	0.838
Cancer			
No	296 (97.37%)	1	
Yes	8 (2.63%)	0.89 (0.22, 3.63)	0.8745
Associated injuries			
No	285 (93.75%)	1	
Yes	19 (6.25%)	0.79 (0.29, 2.17)	0.6079
Dementia			
No	287 (94.41%)	1	
Yes	17 (5.59%)	2.65 (1.33, 5.31)	0.0059
COPD			
No	290 (95.39%)	1	
Yes	14 (4.61%)	0.86 (0.27, 2.71)	0.793
Hepatitis			
No	298 (98.03%)	1	
Yes	6 (1.97%)	0.57 (0.08, 4.10)	0.5769
Gastritis			
No	297 (97.70%)	1	
Yes	7 (2.30%)	0.92 (0.23, 3.73)	0.9031
Treatment strategy			
CRIF/ORIF	211 (69.41%)	1	
HA	91 (29.93%)	0.95 (0.59, 1.53)	0.8365
THA	2 (0.66%)	0.00 (0.00, Inf)	0.9957
Operation time (minutes)	98.48 ± 36.43	1.00 (1.00, 1.01)	0.4812
Blood loss (mL)	229.39 ± 140.51	1.00 (1.00, 1.00)	0.4187
Transfusion (units)	1.03 ± 1.23	1.08 (0.91, 1.28)	0.3909
Infusion (mL)	1,578.79 ± 369.55	1.00 (1.00, 1.00)	0.3085
Time to admission (hours)	113.38 ± 376.39	1.00 (1.00, 1.00)	0.7554
Time to operation (days)	4.68 ± 2.33	0.96 (0.87, 1.06)	0.4592
Length of stay (days)	8.41 ± 2.90	1.03 (0.96, 1.11)	0.3638
UHR	230.59 ± 109.97	1.00 (1.00, 1.00)	0.0213

UHR, Uric acid to high-density lipoprotein cholesterol ratio; CHD, coronary heart disease; COPD, chronic obstructive pulmonary disease; HA, hemiarthroplasty; CRIF/ORIF, closed/open reduction and internal fixation; THA, total hip arthroplasty.

The association between UHR and mortality was evaluated using Cox proportional hazards regression. Restricted cubic spline smoothing was used to inspect the functional form. A single-slope Cox model was then compared with a two-piecewise Cox model using a likelihood-ratio test; the inflection point was estimated from the observed data. Because the threshold and slopes were derived in the same cohort, all nonlinear and threshold findings were treated as exploratory.

All statistical analyses were conducted using R (http://www.R-project.org; The R Foundation) and EmpowerStats (http://www.empowerstats.com; X&Y Solutions Inc., Boston, MA). Results are presented as hazard ratios (HRs) with 95% confidence intervals (CIs), and a two-sided *P* < 0.05 was considered statistically significant. No *a priori* sample-size calculation was performed because this retrospective study included all eligible patients during the study period. With 82 deaths, the fully adjusted continuous-UHR model contained seven regression parameters (approximately 11.7 events per parameter); models using UHR tertiles or two UHR slopes contained eight fixed regression parameters (approximately 10.3 events per parameter), although estimation of a data-driven inflection point adds uncertainty not captured by this simple ratio.

## 3 Results

### 3.1 Patient characteristics

In this study, we included 304 patients with geriatric hip fractures, 104 males and 200 females. The mean age was 80.00 ± 6.50 years. There were 296 falls, 5 accidents, and 3 other injuries in the mechanism. There were 214 intertrochanteric fractures and 90 femoral neck fractures. The mean aCCI was 4.30 ± 1.10. The mean time to admission was 113.38 ± 376.39 h. The mean time to operation was 4.68 ± 2.33 days. There were 211 patients receiving CRIF/ORIF, 91 HA, and 2 THA. The mean operation time was 98.48 ± 36.43 min. The mean blood loss was 229.39 ± 140.51 mL. The mean transfusion volume was 1.03 units.

Patients were divided into sample-based UHR tertiles: low UHR 57.71–166.67, medium UHR 168.91 to < 253.16, and high UHR 254.20–846.34. The corresponding group means were 126.83 ± 26.09 (*n* = 100), 208.27 ± 26.06 (*n* = 101), and 353.22 ± 91.45 (*n* = 103), respectively.

Baseline characteristics are shown in Table 1. The proportion of men increased across UHR tertiles (18.00%, 37.62%, and 46.60%), although women remained the numerical majority in the high-UHR group (55 women vs. 48 men). Fracture classification, CHD prevalence, time to admission, and crude mortality also differed across groups. Time to admission showed a non-monotonic pattern (66.49, 190.73, and 83.06 h in the low-, medium-, and high-UHR groups, respectively); therefore, this difference should not be interpreted as a dose-response relationship or a consequence of UHR.

The mean follow-up period was 34.42 ± 14.76 months. During follow-up, there were 82 all-cause deaths.

### 3.2 Univariable analysis of the association between variables and mortality

Univariable Cox regression results are shown in Table 2. Age, aCCI, CHD, arrhythmia, and dementia had *P* < 0.10 and were therefore considered for adjustment. Sex did not meet the *P* < 0.10 criterion (*P* = 0.1668) but was retained *a priori* because it is a clinically established prognostic factor in older patients with hip fracture. Model II therefore adjusted for age, sex, aCCI, CHD, arrhythmia, and dementia.

### 3.3 Multivariable analysis of preoperative UHR and mortality

In the conventional Cox models shown in Table 3, UHR was associated with mortality only before adjustment. After full adjustment, neither continuous UHR (*P* = 0.1413) nor the medium and high tertiles versus the low tertile (*P* = 0.1344 and *P* = 0.1624, respectively) reached statistical significance, and the adjusted trend test was also non-significant (*P* = 0.1970). These results do not support describing UHR as an independent predictor under a linear or tertile parameterization. The difference between the unadjusted and adjusted estimates, together with the shape of the fitted curve, motivated an exploratory assessment of nonlinearity.

**Table 3:** Multivariable results by Cox regression.

Exposure	Non-adjusted	Adjust I	Adjust II
UHR	1.00 (1.00, 1.00) 0.0213	1.00 (1.00, 1.00) 0.1509	1.00 (1.00, 1.00) 0.1413
UHR tertile			
Low	1	1	1
Medium	1.85 (1.04, 3.32) 0.0372	1.63 (0.90, 2.93) 0.1052	1.58 (0.87, 2.89) 0.1344
High	1.89 (1.06, 3.36) 0.0298	1.47 (0.81, 2.69) 0.2064	1.54 (0.84, 2.84) 0.1624
*P* for trend	0.0349	0.2555	0.1970

Data in the table: HR (95% CI).

Outcome variable: Mortality.

Exposure variables: UHR; UHR tertile; UHR tertile continuous.

Adjust model I adjusted for age, sex.

Adjust model II adjusted for age, sex, aCCI, CHD, arrhythmia, and dementia.

### 3.4 Curve fitting and analysis of the threshold or saturation effect

Figure 1 suggested a nonlinear pattern after adjustment. In the exploratory two-piecewise Cox model, the estimated inflection point was 166.17, as shown in Table 4. Below this point, the estimated slope was positive (HR per unit=1.01, 95% CI 1.00–1.03, *P* = 0.0326); above it, the incremental slope was close to null and not statistically significant (HR per unit=1.00, 95% CI 1.00–1.00, *P* = 0.8050). The likelihood-ratio test comparing the single-slope and two-piecewise models yielded *P* = 0.037. This plateau in the slope does not imply that patients above 166.17 have the same absolute risk as patients with low UHR; it indicates only that risk did not continue to increase detectably with each additional UHR unit above the estimated inflection point.

**Figure 1:**
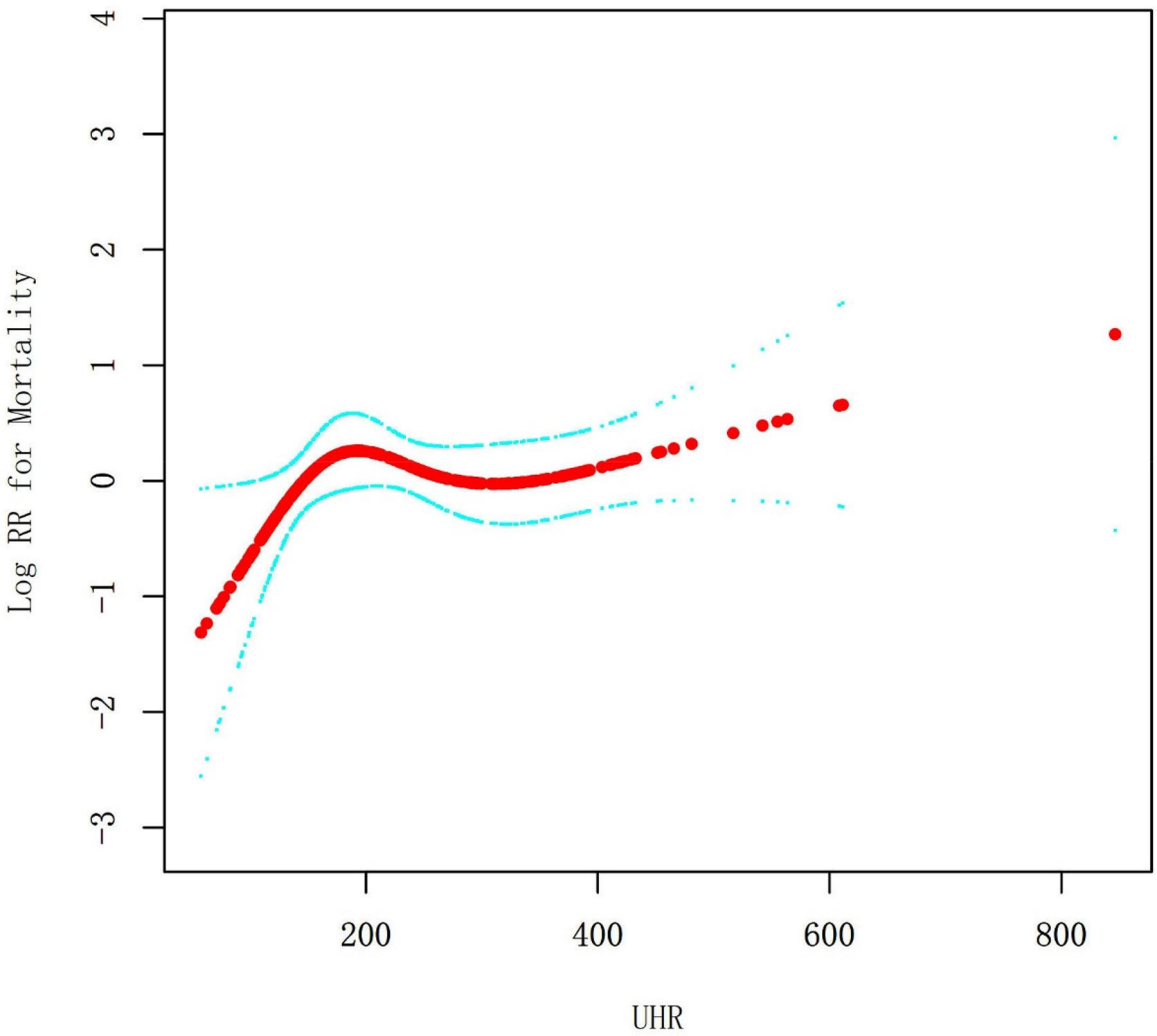
Adjusted smooth curve for the association between admission UHR and all-cause mortality. The red curve shows the estimated log relative hazard, and the two cyan dashed curves show the 95% confidence limits. The model was adjusted for age, sex, aCCI, coronary heart disease, arrhythmia, and dementia. Wider confidence limits at the extremes reflect sparse observations.

**Table 4:** Exploratory comparison of single-slope and two-piecewise Cox models for UHR and all-cause mortality.

Model	Mortality
Cox proportional hazards regression model	1.00 (1.00, 1.00) 0.1413
The two-piecewise Cox proportional hazards regression model	
Inflection point (K)	166.17
<K	1.01 (1.00, 1.03) 0.0326
>K	1.00 (1.00, 1.00) 0.8050
*P* value for the log-likelihood ratio test	0.037

Values are HR (95% CI) and *P* value. The likelihood-ratio test compares the fit of the two-piecewise model with the nested single-slope Cox model; it is not a test conducted only within the two-piecewise model.

Outcome variable: Mortality.

Exposure variable: UHR.

Adjusted variables: age, sex, aCCI, CHD, arrhythmia, dementia.

### 3.5 Kaplan-Meier survival

Figure 2 shows Kaplan–Meier survival curves for the lower-UHR group (<166.17) and higher-UHR group (≥166.17). The higher-UHR group had lower unadjusted survival during follow-up. Because 166.17 was selected from the same dataset, this dichotomized comparison is descriptive and requires external validation.

**Figure 2:**
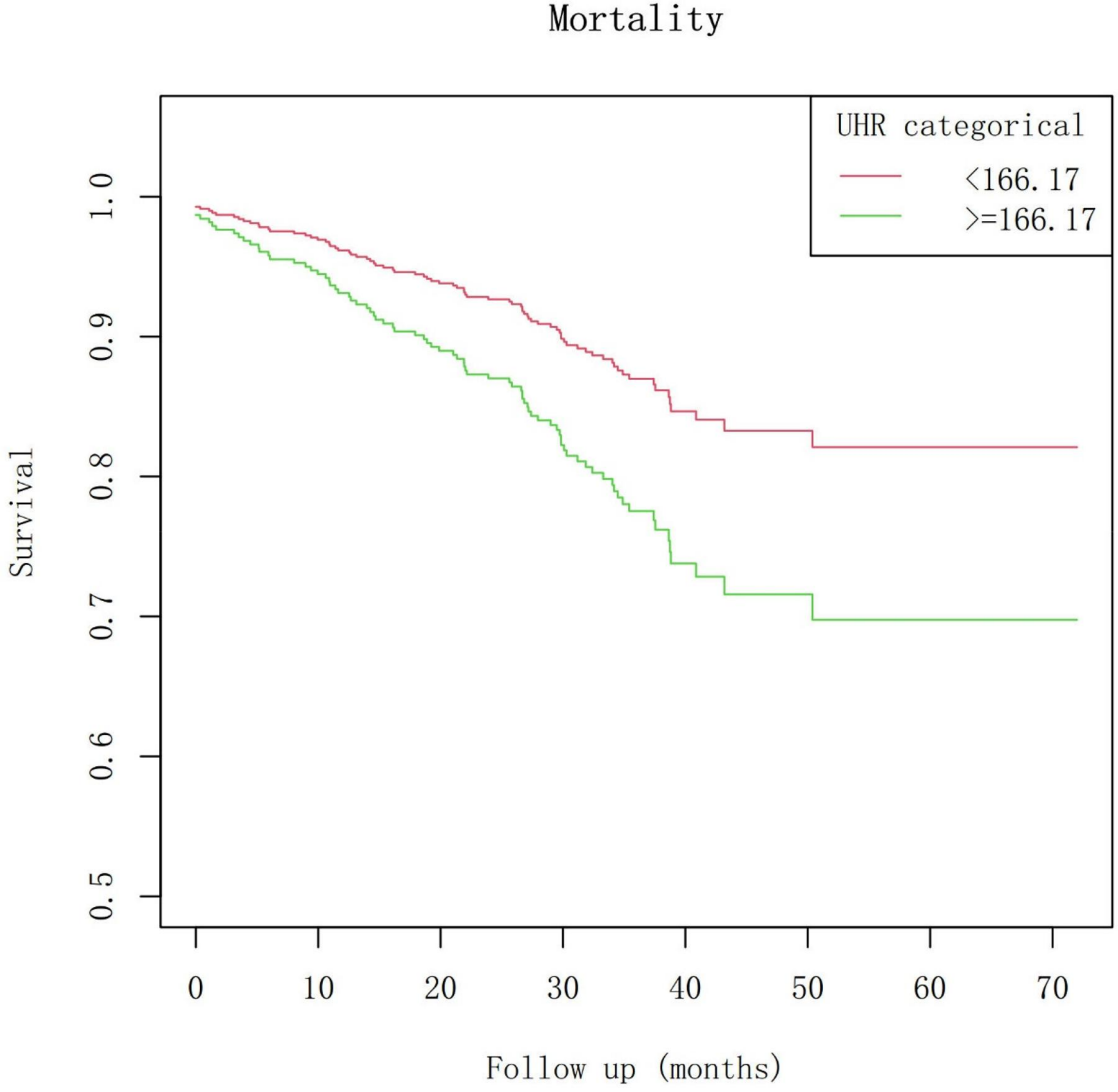
Kaplan–Meier survival curves stratified by the exploratory UHR threshold. The red curve represents the lower-UHR group (UHR <166.17), and the green curve represents the higher-UHR group (UHR ≥166.17). This unadjusted grouping uses a data-derived threshold and should not be interpreted as a validated clinical classification.

## 4 Discussion

The main finding was an exploratory nonlinear association between admission UHR and long-term all-cause mortality. Conventional fully adjusted analyses of continuous UHR and UHR tertiles were not statistically significant. The two-piecewise model suggested that the slope was positive below an estimated inflection point of 166.17 and approximately flat above it. We use the term plateau to describe this fitted shape, not to imply a proven biological saturation mechanism. The likelihood-ratio test favored the two-piecewise over the single-slope model (*P* = 0.037), but the data-derived threshold and modest number of deaths require cautious interpretation and external validation.

The baseline findings should also be interpreted descriptively. The proportion of men increased across UHR tertiles, but women still outnumbered men in the high-UHR group (55 vs. 48). Time to admission differed among tertiles but was not monotonic, making a causal explanation inappropriate. Crude mortality was higher in the high- than low-UHR tertile (32.04% vs. 18.00%); this group-level difference is compatible with a plateau above the inflection point because a null slope above 166.17 means that additional increases within the higher range were not detectably associated with further risk. It does not erase risk differences accumulated across lower UHR values. Nevertheless, the fully adjusted tertile comparison was not significant, so UHR cannot be described as an independent predictor on the basis of these data.

Prior studies have related UHR to cardiovascular and mortality outcomes, but reported functional forms and thresholds vary across populations. A US population analysis reported a nonlinear threshold of 12.4% for all-cause mortality when UHR was calculated as uric acid (mg/dL)/HDL-C (mg/dL) × 100 (22). Under the unit convention used in the present study, 166.17 μmol/mmol corresponds to approximately 7.22% after conversion to mg/dL/mg/dL × 100. These values should not be treated as directly interchangeable because study populations, outcomes, covariate adjustment, and threshold-selection methods differ. In hip-fracture prognosis, several complementary domains have been investigated. Lower hip muscle size and density were independently associated with mortality in a prospective cohort of older patients with hip fracture (23). Preoperative frailty assessed using the FRAIL scale was associated with higher 1-year mortality after hip fracture surgery (24). The prognostic nutritional index, derived from serum albumin and lymphocyte count, was associated with postoperative complications and 2-year all-cause mortality in patients with hip fracture (25). These findings indicate that UHR should be evaluated as a potentially complementary metabolic marker rather than a replacement for established clinical, frailty, muscle, inflammatory, or nutritional assessments.

UHR is inexpensive and can be calculated from routine laboratory measurements, but convenience alone does not establish clinical usefulness. Our data support only a hypothesis-generating nonlinear association. The value 166.17 is a cohort- and unit-specific estimated inflection point rather than a validated clinical benchmark, and patients with values above this point should not automatically receive intensified treatment on the basis of UHR alone. Future studies should test whether UHR adds prognostic information to validated clinical, frailty, sarcopenia, inflammatory, and nutritional measures and should evaluate calibration and decision-analytic benefit before any cutoff is used in practice.

Several biological pathways could plausibly link a high admission UHR with adverse outcomes, including oxidative stress, inflammation, endothelial dysfunction, and reduced HDL-related antioxidant capacity (26–30). However, the present study did not measure inflammasome activation, cytokines, oxidative injury, or HDL function. These mechanisms are therefore speculative and should be regarded only as possible explanations to be tested in mechanistic studies, not as conclusions supported by our data.

This study has several limitations. First, it was a single-center retrospective analysis with 304 participants and 82 deaths, which limits generalizability and statistical precision. Conventional events-per-parameter ratios were approximately 11.7 for the fully adjusted continuous-UHR model and 10.3 for models with UHR tertiles or two UHR slopes; however, selection of the inflection point from the same data adds model complexity and increases the possibility of optimism and overfitting. The threshold analysis should therefore be considered exploratory. Second, UHR was measured once after admission for an acute hip fracture. Trauma, fasting status, hydration, renal function, medications, and perioperative stress may alter uric acid or HDL-C, so this value may not represent the pre-fracture metabolic state. Third, only all-cause mortality was available. A competing-risk model is not applicable when every death is counted as the event, but the absence of adjudicated cause-specific mortality prevented evaluation of cardiovascular, infectious, malignant, or other causes of death. Fourth, residual confounding from lifestyle, medication, renal function, frailty, sarcopenia, and socioeconomic factors remains possible. Finally, the study did not evaluate whether UHR improves discrimination, calibration, or clinical decisions beyond established risk measures, and the data-derived inflection point requires external prospective validation.

## 5 Conclusion

In this retrospective cohort, admission UHR showed an exploratory nonlinear association with long-term all-cause mortality. Conventional fully adjusted analyses were not statistically significant. A two-piecewise model suggested a positive slope below a data-derived inflection point of 166.17 and no detectable additional increase above that point. These findings do not establish UHR as an independent predictor or 166.17 as a clinical cutoff.

Prospective multicenter studies should confirm the functional form of the association, repeat UHR measurements after the acute stress period, validate any threshold externally, and determine whether UHR adds prognostic value beyond clinical, frailty, sarcopenia, inflammatory, and nutritional measures.

## Data Availability

The raw data supporting the conclusions of this article will be made available by the authors, without undue reservation.
